# Genome Wide Analysis Approach Suggests Chromosome 2 Locus to be Associated with Thiazide and Thiazide Like-Diuretics Blood Pressure Response

**DOI:** 10.1038/s41598-019-53345-5

**Published:** 2019-11-21

**Authors:** Sonal Singh, Caitrin W. McDonough, Yan Gong, Kent R. Bailey, Eric Boerwinkle, Arlene B. Chapman, John G. Gums, Stephen T. Turner, Rhonda M. Cooper-DeHoff, Julie A. Johnson

**Affiliations:** 1 Department of Pharmacotherapy and Translational Research and Center for Pharmacogenomics, University of Florida, Gainesville, Florida USA; 2 Division of Biostatistics, Department of Health Sciences Research, Mayo Clinic, Rochester, Minnesota USA; 3 Human Genetics and Institute of Molecular Medicine, University of Texas Health Science Center, Houston, Texas USA; 4 Division of Nephrology, University of Chicago, Chicago, IL USA; 5 Division of Nephrology and Hypertension, Mayo Clinic, Rochester, Minnesota USA; 6 Division of Cardiovascular Medicine, Department of Medicine, University of Florida, Gainesville, Florida USA

**Keywords:** Genome-wide association studies, Hypertension

## Abstract

Chlorthalidone (CTD) is more potent than hydrochlorothiazide (HCTZ) in reducing blood pressure (BP) in hypertensive patients, though both are plagued with BP response variability. However, there is a void in the literature regarding the genetic determinants contributing to the variability observed in BP response to CTD. We performed a discovery genome wide association analysis of BP response post CTD treatment in African Americans (AA) and European Americans (EA) from the Pharmacogenomic Evaluation of Antihypertensive Responses-2 (PEAR-2) study and replication in an independent cohort of AA and EA treated with HCTZ from the PEAR study, followed by a race specific meta-analysis of the two studies. Successfully replicated SNPs were further validated in beta-blocker treated participants from PEAR-2 and PEAR for opposite direction of association. The replicated and validated signals were further evaluated by protein-protein interaction network analysis. An intronic SNP rs79237970 in the *WDR92* (eQTL for *PPP3R1*) was significantly associated with better DBP response to CTD (p = 5.76 × 10^−6^, β = −15.75) in the AA cohort. This SNP further replicated in PEAR (p = 0.00046, β = −9.815) with a genome wide significant meta-analysis p-value of 8.49 × 10^−9^. This variant was further validated for opposite association in two β-blockers treated cohorts from PEAR-2 metoprolol (p = 9.9 × 10^−3^, β = 7.47) and PEAR atenolol (p = 0.04, β = 4.36) for association with DBP. Studies have implicated *WDR92* in coronary artery damage. *PPP3R1* is the regulatory subunit of the calcineurin complex. Use of calcineurin inhibitors is associated with HTN. Studies have also shown polymorphisms in *PPP3R1* to be associated with ventricular hypertrophy in AA hypertensive patients. Protein-protein interaction analysis further identified important hypertension related pathways such as inositol phosphate-mediated signaling and calcineurin-NFAT signaling cascade as important biological process associated with PPP3R1 which further strengthen the potential importance of this signal. These data collectively suggest that *WDR92* and *PPP3R1* are novel candidates that may help explain the genetic underpinnings of BP response of thiazide and thiazide-like diuretics and help identify the patients better suited for thiazide and thiazide-like diuretics compared to β-blockers for improved BP management. This may further help advance personalized approaches to antihypertensive therapy.

## Introduction

Thiazides and thiazide-like diuretics remain a recommended first line therapy for treating uncomplicated hypertension^1,2^. Hydrochlorothiazide (HCTZ) and chlorthalidone (CTD) are the most commonly prescribed drugs of these classes. The guidelines however do not distinguish one thiazide over the other as a first line option for hypertension management^3^. Even though most of the diuretic HTN clinical trials with mortality end points have used chlorthalidone^4–6^,over the years, HCTZ has been the more commonly prescribed thiazide. However, CTD is gaining favor over HCTZ given the fact that several studies have reported greater benefits associated with the use of CTD compared to HCTZ for hypertension management^7^. There are distinct differences between the pharmacokinetic profiles of the two drugs as well with CTD being more potent and having a longer duration of action as compared to HCTZ^8,9^. Overall, these studies indicate that there may be additional benefits of using CTD compared to HCTZ.

**Figure 1:**
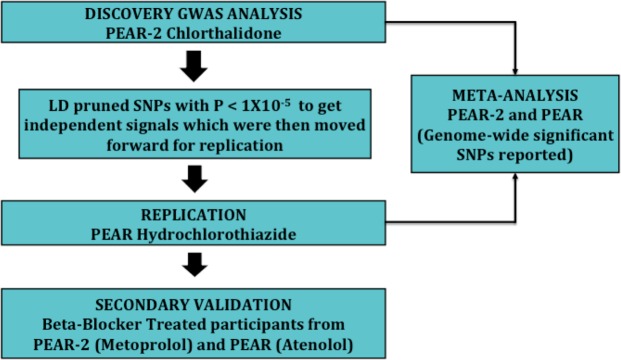
Overall steps for the multistage GWAS analysis approach.

CTD use for hypertension management however is associated with wide variability in blood pressure (BP) response as is true for all classes of antihypertensive medications^10^. The increasing interest in CTD for hypertension management makes the *a priori* identification of patients more suited for CTD therapy very important for better outcomes and BP control. However, there is a paucity of literature regarding the effect of genetics on the variability observed in CTD BP response. To this end, we undertook a multi-stage genome wide analysis approach (Fig. 1) to identify the genetic underpinnings of the CTD BP response. To our knowledge, this is the first genome wide association study undertaken to identify the pharmacogenomic markers that may help explain the BP variability observed with CTD treatment.

## Results

The demographics and clinical characteristics of the participants of PEAR-2 and PEAR are presented in Table 1. Age and baseline BP was similar across the PEAR-2 and PEAR cohorts in both the race groups. CTD is a more potent antihypertensive compared to HCTZ, which explains DBP and SBP responses being significantly higher for the CTD group compared to HCTZ for both European and African American race groups.

**Table 1:** Clinical Characteristics of PEAR-2 and PEAR participants.

Clinical Characteristics	PEAR-2 (Chlorthalidone)		PEAR (Hydrochlorothiazide)	
	African Americans (N = 142)	European Americans (N = 175)	African Americans (N = 148)	European Americans (N = 222)
Age, years	50.01 ± 8.81	51.22 ± 8.82	47.38 ± 8.82*	50.16 ± 9.46
Female, N (%)	65 (48.14)	75(42.85)	92(62.16)	89(40.09)
Baseline SBP	146.49 ± 11.06	147.48 ± 10.34	147.08 ± 11.54	151.06 ± 13.41
Baseline DBP	94.88 ± 5.99	94.31 ± 5.04	95.06 ± 6.56	97.05 ± 5.24*
SBP Response	−15.07 ± 10.07	−12.25 ± 9.1	−11.97 ± 9.72*	−8.50 ± 7.02*
DBP Response	−8.56 ± 6.26	−6.83 ± 5.43	−7.11 ± 6.47*	−4.68 ± 4.79*

Values are presented as mean ± standard deviation unless otherwise noted. PEAR:

Pharmacogenomic Evaluation of Antihypertensive Response.

^*^Indicates P < 0.05 for chlorthalidone (PEAR-2) vs Hydrochlorothiazide (PEAR) within race.

### Genome wide association analysis

Genome wide association analysis was carried out to test the association between the variants and change in diastolic and systolic BP response post treatment with CTD. The Manhattan plots for discovery association with DBP and SBP response post CTD treatment are presented in Supplementary Fig. 1. Post genome wide association analysis, none of the SNPs met genome wide significance for association with either DBP or SBP response in either of the race groups. However, 298 SNPs and 107 SNPs meet the suggestive level of significance (p < 1 × 10^−5^) for association with change in DBP and SBP respectively in the African American race group. In the European American race group, 103 SNPs were associated with change in DBP and 78 SNPs were associated with the change in SBP response respectively. These SNPs were LD pruned (r2 > 0.8) to get one independent variant representing each gene locus which was then tested for replication. Post pruning, in the African American cohort 72 SNPs for association with DBP (Supplementary Table 1) and 36 SNPs for association with SBP (Supplementary Table 2) and in the European American cohort, 29 SNPs and 23 SNPs for association with DBP (Supplementary Table 3) and SBP response (Supplementary Table 4) respectively were moved forward for replication. Based on the Bonferroni correction for multiple testing, the replication threshold was set at p-value 6.9 × 10^−4^ for association with change in DBP and at p < 1.3 × 10^−3^ for association with change in SBP in the African American cohort and at p < 1.7 × 10^−3^ and at p < 2.1 × 10^−3^ for DBP and SBP in the European American cohort respectively. The independent signals from the discovery were tested for replication in the HCTZ treated participants from PEAR.

**Figure 2:**
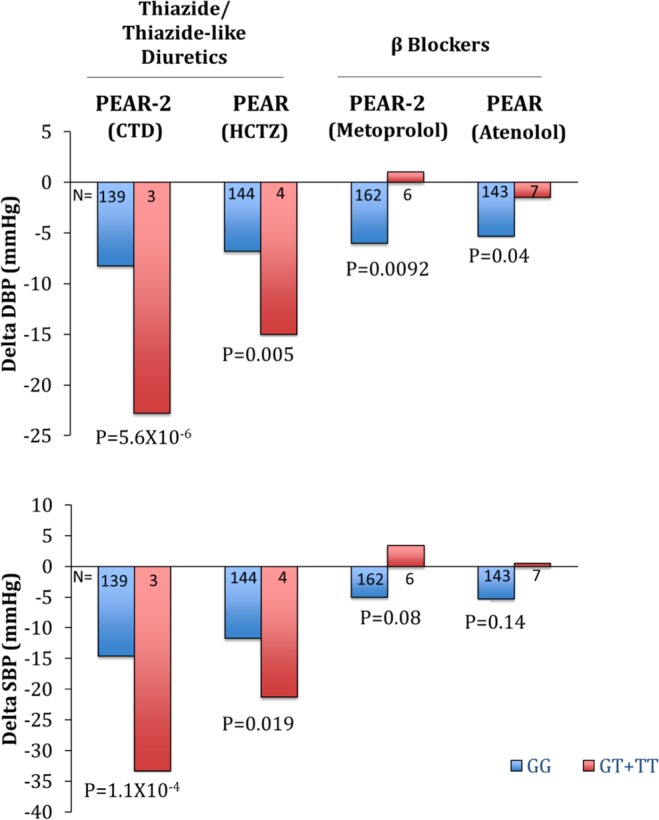
Blood Pressure change response by genotype of rs79237970 for the chlorthalidone, hydrochlorothiazide, metoprolol and atenolol treated participants from PEAR-2 and PEAR.

Of the SNPs taken forward for replication, one SNP rs79237970 met the Bonferroni correction for replication (p = 0.00046, β = −9.81) for association with change in DBP response in the African American race group (Table 2A). Rs79237970 is present in the *WDR92* and is also an eQTL for the *PPP3R1* gene. Rs79237970 was imputed with high quality in both PEAR-2 and PEAR (Rsq = 0.83) datasets. However, given the low minor allele frequency of the SNP in the PEAR datasets (MAF = 0.013) we verified the concordance between the genotyped and the imputed rs79237990 and found 100 percent concordance between the two. Because of the low frequency we did not have any homozygote variant carriers in PEAR-2 and had one homozygote carrier in PEAR. Hence, we also ran a dominant model by comparing variant carriers versus non-carriers and testing the association for change in BP response. Like the additive model, the T allele carriers had better BP response (both DBP and SBP response) compared to the G allele carriers in both the PEAR-2 and PEAR studies (Fig. 2).

**Table 2:** Association results of the replicated SNP rs79237970 in the CTD and HCTZ treated (A) and βB treated (B) African American participants.

SNP	CHR	A1	MAF	DISCOVERY PEAR-2 Chlorthalidone		REPLICATION PEAR1 Hydrochlorothiazide		Meta-Analysis (PEAR-2 and PEAR)	
				β	P-value	β	P-value	β	P-value
(**A) Association of rs79237970 with DBP in CTD and HCTZ treated African American participants**									
rs79237970	2	T	0.013	−15.75	5.76 × 10^−6^	−9.815	0.0004	−12.02	8.49 × 10^−9^
**(B) Association of rs79237970 with DBP in βB treated African American participants**									
**SNP**	**CHR**	**A1**	**MAF**	**PEAR-2** **Metoprolol**		**PEAR** **Atenolol**		**Meta-Analysis** **(PEAR-2 and PEAR)**	
				**β**	**P-value**	**β**	**P-value**	**β**	**P-value**
rs79237970	2	T	0.013	7.47	9.9 × 10^−3^	4.36	0.04	5.6	1.5 × 10^−3^

CHR: chromosome; A1: Effect Allele; MAF: Minor Allele Frequency; **β**: regression coefficient for Effect Allele; DBP: Diastolic

Blood Pressure; CTD: Chlorthalidone; HCTZ: Hydrochlorothiazide.

Unfortunately, none of the SNPs met the Bonferroni threshold for replication for association with SBP in the African American race group and for either DBP or SBP response in the European American race group.

### Validation

We further validated the replicated SNP rs79237970 for significant association in the opposite direction as that of thiazide in the metoprolol and atenolol treated African Americans cohorts from PEAR-2 and PEAR respectively. rs79237970 was successfully validated in both the βB cohorts, as it was significantly associated with DBP response with opposite direction of association in both the PEAR-2 metoprolol (p = 9.9 × 10^−3^, β = 7.47) and PEAR atenolol (p = 0.04, β = 4.36) treated participants from PEAR-2 and PEAR respectively (Table 2B).

### Meta-analysis

The results from PEAR-2 and PEAR were further combined into race specific meta-analysis and we report the genome wide significant SNPs for association with DBP and SBP (Table 3). In the African American cohort, for association with change in DBP response, the replicated SNP, rs79237970 in the *WDR92* reached a genome wide significant p-value of 8.49 × 10^−9^, and for association with change in SBP, rs79944011 present 38 kb 5′ of *SLC35B4* reached genome wide significance (p = 4.9 × 10^−08^). In the European American cohort, for association with SBP, rs1442118 (1.73 × 10^−08^) present in the *RAB9BP1* reached genome wide significance and for association with DBP, three SNPs rs118131161 (1.11 × 10^−08^) in the *COL4A1*, rs2400940 (4.26 × 10^−08^) in the *DLK1* and rs118140307 (4.83 × 10^−08^) in *LRRC4C* reached genome wide significance.

**Table 3:** Genome wide significant SNPs from race specific meta-analysis of PEAR-2 (Chlorthalidone treated) and PEAR (Hydrochlorothiazide) treated participants.

SNP	CHR		Nearest Gene	A1	MAF	Phenotype	Meta-Analysis	
							β	P-value
**African Americans**								
rs79237970		2	*WDR92*	T	0.013	DBP	−12.02	8.49 × 10^−9^
rs79944011		7	*SLC35B4*	A	0.012	SBP	19.13	4.97 × 10^−08^
**European Americans**								
rs118131161		13	17 kb 3′ of *COL4A1*	G	0.024	DBP	7.3599	1.11 × 10^−08^
rs2400940		14	21 kb 5′ of *DLK1*	T	0.18	DBP	2.5285	4.26 × 10^−08^
rs118140307		11	*LRRC4C*	C	0.03	DBP	7.1643	4.83 × 10^−08^
rs1442118		5	745 kb 5′ of *RAB9BP1*	A	0.38	SBP	3.89	1.73 × 10^−08^

CHR: chromosome; A1:Effect Allele; MAF: Minor Allele Frequency; β: regression coefficient for Effect Allele.

### Protein-protein interaction network analysis

**Figure 3:**
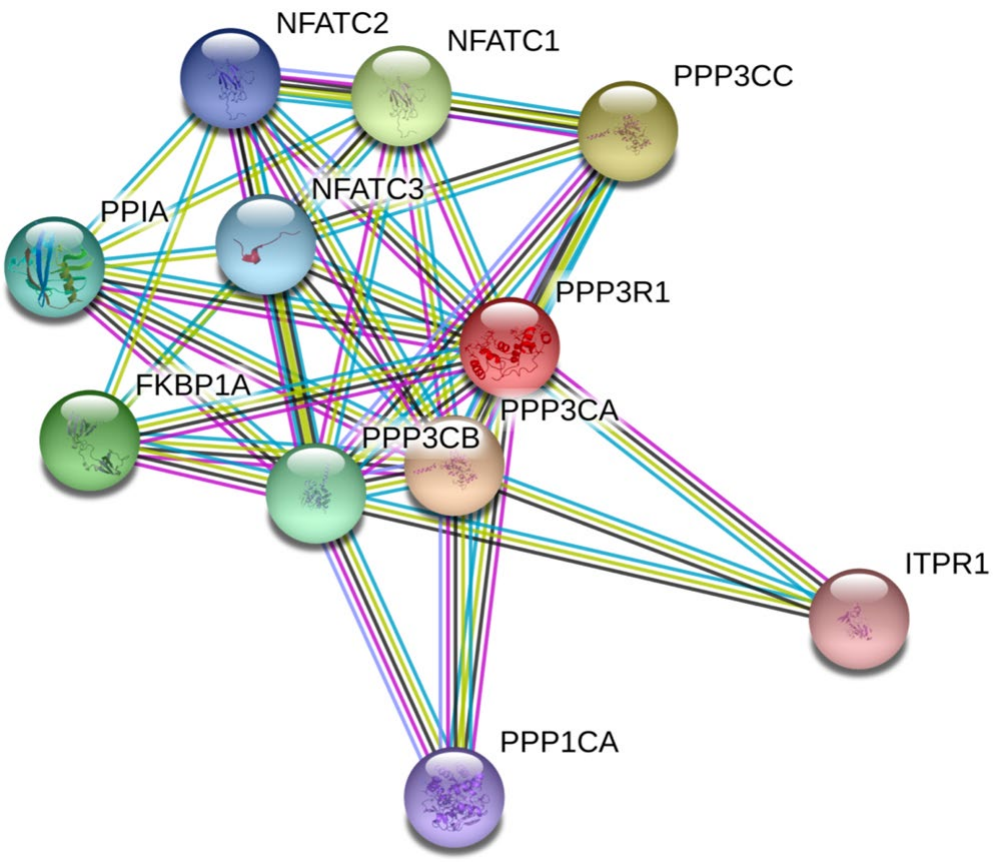
Protein-protein interaction network for PPP3R1 depicting important protein interactions with other components of calcineurin complex and NFAT proteins.

To better understand the mechanistic underpinnings of the replicated and validated genes discovered in this study (PPP3R1 and WDR92), we performed a protein-protein interaction network analysis. For *PPP3R1*, using a high STRING score threshold, we identified direct interactions between PPP3R1 and other subunits of calcineurin (PPP3CB, PPP3CC and PPP3CA). Interestingly, we also found interactions between PPP3R1 and NFATC1 and NFATC3 (Fig. 3). The most significant biological process identified for the protein-protein interaction network by STRING are reported in Supplementary Table 5. Biologically, inositol phosphate-mediated signaling (Gene Ontology:0048016) (FDR = 1.98 × 10^−15^) and calcineurin-NFAT signaling cascade (Gene Ontology:0033173) (FDR = 1.99 × 10^−14^) have significant literature support for involvement in hypertension related signaling mechanisms. The STRING database did not identify any significant interactions for *WDR92*.

## Discussion

Hypertension is one of the most common chronic diseases for which medications are prescribed and thiazide diuretics are a recommended first line therapy for treating uncomplicated hypertension. Among the thiazides, even though a head to head comparison has not been made, clinical trial data collectively suggest CTD might be superior to HCTZ for BP management and associated with better outcomes^7^. Though several attempts have been made to investigate the underlying genetic determinants for HCTZ BP response^11–14^, to our knowledge this is the first genome wide association analysis assessing the influence of pharmacogenomic variants on CTD BP response.

Using a multi-stage GWAS approach we identified a variant rs79237970 in the *WDR92* that was significantly associated with diastolic BP response post CTD treatment among the African American population in PEAR-2. We successfully replicated this association using an independent cohort of HCTZ treated African American participants from PEAR. Several studies have established the opposite association of βB and thiazide BP response given the opposite mechanism of actions of these two antihypertensive classes with respect to the renin angiotensin system wherein βBs inhibit the renin whereas thiazide causes activation of renin^15^. Using this theory for secondary validation, we showed a significant, opposite direction response to this SNP for both metoprolol and atenolol. The variant carriers had increased BP response to thiazide treatment and poor BP response to βB treatment compared to the non-carriers. It is important to identify patients better suited for one therapy versus the other for optimum BP management and the data suggest this variant may help identify patients who would respond well to a thiazide and poorly to a βB.

Rs79237970 is present in the *WDR92* and is eQTL for *PPP3R1* in the chr2p14 region of the chromosome 2. We found several pieces of evidence in the literature in support of the genes in this region with respect to their involvement with BP modulation. A recent report by Evangelou *et al*., used about one million people to identify genetic loci that are associated with BP using the UKbiobank and International Consortium for Blood Pressure (ICBP) data. They identified *WDR92* as one of the novel loci that was associated with BP^16^. Specifically with respect to *PPP3R1*, HYPER-GEN sub study found a novel 5 base pair deletion in the *PPP3R1* promoter region to be significantly associated with left ventricular hypertrophy in severe hypertensive African American patients^17^. *PPP3R1* encodes for the regulatory subunit of calcineurin, which is a Ca^2+^/calmodulin-activated serine/threonine protein phosphatase and is critical for a variety of pathways, including angiogenesis, calcium homeostasis and hypertrophy signaling^18–20^. The use of calcineurin inhibitors as immunosuppressive agents is associated with development of hypertension. Studies have shown a significant inverse correlation between the calcineurin and the renin system wherein inhibition of calcineurin results in increased renin levels due to the activation of renin angiotensin system^21,22^. Rs79237970 is an eQTL for *PPP3R1* with the variant carriers having increased expression of the *PPP3R1* compared to non-carriers (Supplementary Fig. 1). Increased renin activity has also been associated with better BP response to βBs and lesser response to thiazides^15^. Thus, increased expression of PPP3R1 in variant carriers may lead to decreased renin activity, which may be the underlying mechanism of rs79237970 variant carriers having better BP response to thiazide and decreased BP response to βBs observed in our study. Collectively these data indicate the involvement of rs79237970 and the *WDR92/PPP3R1* region with RAS pathway mediated BP regulation.

To understand the possible implication of the genetic variance in *PPP3R1* we further built a protein-protein network analysis using known interactions with the highest level of confidence to further investigate the molecular complexes and processes that PPPR1 interacts with. PPP3R1 interacts with the other catalytic subunits of calcineurin, which indicates that genetic perturbation in PPP3R1 can have functional effects for the entire calcineurin complex. Another important interaction of PPP3R1 was with NFAT molecules and likewise the major biological process that PPP3R1 is significantly associated with was the Calcineurin-NFAT signaling cascade, which is implicated in a variety of pathways^23^. With respect to hypertension, NFAT-eNOS signaling pathway plays an important role in regulating normal BP^24^. Given these intricate cross talks and overlaps between the calcineurin pathways, hypertension signaling and the association of calcineurin, renin and antihypertensive medications, the elucidation of the exact mechanisms of WDR92/PPP3R1 region is warranted.

In addition, with increased power by combining PEAR-2 and PEAR thiazide cohorts in a race specific meta-analysis, we identified additional genome wide signals. We identified variants in the *RAB9BP1* for association with SBP and for DBP response; we identified variants in *COL4A1, DLK1 and LRRC4C* that reached genome wide significance in the European American cohort. Of these, studies in mice have shown that mutations in the *COL4A1* has been associated with low BP because of defective vasculature^25^. In a conditional analysis looking at the genetics of bone mineral density and other pleiotropic phenotypes, *LRRC4C* was significantly associated with DBP^26^. In the African American cohort, for association with SBP response, rs79944011 that is 38 kb 5′ of *SLC35B4* reached genome wide significance. Yazbek *et al*., have found *SLC35B4* to be a potential regulator of obesity and glucose homeostasis which are important predictors of hypertension^27^. These data suggest the possible implications of these regions in the thiazide mediated BP regulation. However further independent replication of these signals and detailed studies investigating the underlying mechanisms are needed to completely understand their involvement in CTD BP response.

The current study has several strengths. Namely, to our knowledge this is the first genome wide association study investigating the underlying genetic markers associated with CTD BP response. Furthermore, we not only successfully replicated the signal in *PPP3R1* in an independent HCTZ cohort but further validated it in two βB treated cohorts as well. Lastly, we garnered further evidence using protein-protein network analysis that identified interactions and biological processes that have been implicated in hypertension signaling.

We acknowledge the limitations of our study as well. Lack of a distinct replication cohort of CTD treated participants is one of our limitations. Also, given the low minor allele frequency of the SNP, lack of power was our biggest limitation. However, we used a drug of the same class for replication and further used two other βB treated cohorts for validation. We have also provided additional evidence in the form of protein interactions in support of our finding. However further replication of this finding in a larger independent cohort is needed to further confirm this association.

In conclusion, using multi-stage GWAS approach, we identified variant in the *WDR92/PPP3R1* region with significant implication for thiazide BP control and further elucidation of the exact mechanism can ultimately aid in improving individualized antihypertensive BP management.

## Methods

All the data for the Pharmacogenomic Evaluation of Antihypertensive Responses (PEAR) study has been made publicly available in the dbGaP database under dbGaP Accession: phs000649.v1.p1 and can be accessed at (https://www.ncbi.nlm.nih.gov/gap/)^28^. The PEAR-2 data is currently in the process of being uploaded to dbGaP and will soon be available to researchers under Accession: phs000649.v2.p1.

### PEAR-2 and PEAR study design

PEAR-2 and PEAR studies were conducted to investigate the genetic determinants contributing to the variability in the BP response and adverse metabolic effects. The details of both the PEAR studies have been published previously^29^^,^^30^. PEAR-2 was a prospective multicenter trial wherein hypertensive participants were randomized to sequential monotherapy with metoprolol and CTD (clinicaltrials.gov identifier NCT01203852) and PEAR was a prospective multicenter trial where participants were randomized to monotherapy with HCTZ and atenolol (clinical trials.gov identifier NCT00246519), followed by add-on therapy of the alternate drug. Both studies the included participants of either gender and of any race and ethnicity with mild to moderate uncomplicated HTN. Patients with secondary HTN, known history of cardiovascular disease or diabetes were excluded. Eligible participants with uncomplicated HTN underwent an antihypertensive medication washout period of 3–4 weeks. Following confirmation of BP eligibility based on both home and office BP measurements, participants were treated with study specified antihypertensive drugs and doses.

In PEAR, patients were first treated with either atenolol (50 mg daily) or HCTZ (12.5 mg daily) monotherapy for 3 weeks followed by up titration to 100 and 25 mg respectively for a total period of 9 weeks. In PEAR-2, eligible participants were treated with metoprolol tartrate (50 mg twice daily) for two weeks followed by up- titration to 100 mg twice daily for a total period of 8 weeks. The participants were then had an additional washout period again followed by treatment with CTD (15 mg daily) followed up titration to 25 mg daily for a total period of 9 weeks. In both the PEAR-2 and PEAR studies, all patients with a BP of >120/70 mmHg had their dose titrated. At the end of washout (baseline) and following the 8-9-week treatment period in both studies, blood samples were collected for DNA, RNA, plasma and serum, along with measurement of BP.

Both PEAR studies were reviewed and approved by the Institutional Review Boards at the participating sites (University of Florida in Gainesville, FL; Mayo Clinic in Rochester, MN; and Emory University in Atlanta, GA). All participants provided voluntary, written informed consent, and the studies were conducted in accordance with the principles outlined in the Declaration of Helsinki.

### BP response phenotype

For both PEAR-2 and PEAR studies, a change in BP from the start to the end of the antihypertensive treatment (Posttreatment BP – Pretreatment BP) was defined as the diastolic and systolic BP response.

Regarding the BP measurement, the most accurate measurement available for each study was chosen for the analysis. PEAR used a composite weighted average of the office, home and ambulatory since it had higher signal to noise ratio^31^. For PEAR-2 however, only home and office BP measurements were collected and home BP which is the average of BP taken upon before and after going to bed on at least 5 of the previous 7 days was used for the analysis described herein^31^.

### Genotyping and imputation

The details of the genotyping and imputation performed on PEAR-2 and PEAR samples have been previously published^32^. PEAR-2 and PEAR participants were genotyped using the Illumina Human Omni 2.5 S Beadchip (Illumina, San Diego, CA, USA) and Illumina Human Omni1M Quad Beadchip (Illumina, San Diego, CA, USA) respectively. Standard quality control (QC) procedures were applied to both genetic datasets. A principal component analysis was performed using the EIGENSTRAT^33^ method to determine the genetic ancestry of PEAR-2 and PEAR participants. The high-quality SNPs obtained following the QC steps were imputed to the 1000 genomes phase3 version 5 reference panel using Minimac3 (Version 1.0.16)^34^. For post-imputation QC, SNPs with imputation quality (Rsq) <0.3 and minor allele frequency <1% were excluded.

### Statistical analysis

The clinical characteristics of the participants are presented as mean ± standard deviations for the continuous variable. The categorical variables are presented as numbers and percentages. The clinical characteristics were compared within race groups for PEAR-2 and PEAR using a t-test. Genome wide association analysis was carried out to test the association of genetic variants with the change in DBP and SBP response post CTD treatment in both European American as well as African American cohorts. Discovery analysis was carried out using the CTD treated PEAR-2 cohort and PEAR cohort participants treated with HCTZ were used for replication. Beta-blocker (**βB**) treated participants from PEAR-2 and PEAR were used for secondary validation with the assumption of opposite direction responses (i.e. those who respond well to a thiazide respond poorly to a βB. The overall analysis framework is represented in Fig. 1. Post analysis, locus zoom was used to display the regional plot of the association results. AFR for used for the African ancestry group and CEU was used for the European ancestry group^35^.

- **Discovery GWAS:** For both ancestry groups comprised of 142 African Americans and 175 European Americans from the PEAR-2 cohort, the association between the SNPs and the BP response to CTD was tested using linear regression adjusting for age, gender, baseline BP and principal components 1 and 2. Additive mode of inheritance was assumed for the regression analysis. Post analysis, genome wide significance was defined as 5 × 10^−8^ and a suggestive threshold was defined as 1 × 10^−5^. SNPs meeting the suggestive level of significance were LD pruned using LD link^36^ (r2 > 0.8) to obtain single independent SNPs as representative of each locus. The number of independent SNPs was used to determine the Bonferroni threshold for replication (0.05/# of SNPs tested) for both DBP and SBP response.
- **Replication:** Separately amongst the European American and African American cohorts, the independent SNPs from the discovery analysis meeting the suggestive level of threshold were further tested for replication using the HCTZ treated participants from PEAR for DBP and SBP response. The HCTZ treated group was comprised of 148 participants in the African American cohort and 222 participants in the European American cohort. SNPs meeting the Bonferroni threshold in the same direction of association as the discovery were considered replicated.
- **Secondary Validation:** Thiazides and βB have contrasting mechanisms with respect to the renin angiotensin system. We thus postulated that any markers within this pathway significantly associated with the thiazide cohort may be significantly associated with the βB response in the opposite direction. Any SNPs that were successfully replicated in the previous step were hence further tested for validation in the Atenolol treated participants of PEAR and Metoprolol treated participants of PEAR-2. SNPs associated meeting the Bonferroni corrected p-value (0.05/# of SNP tested) with the opposite direction of association as that of thiazides would be considered validated.
- **Meta-analysis:** To increase with the aim of identifying additional variants, the summary statistics of discovery and replication were further combined in race specific meta-analysis between PEAR and PEAR-2. Meta‐Analysis Helper (METAL) was used to perform the meta-analysis using a fixed‐effect model and inverse variance weighing^37^. SNPs meeting genome wide significance (p < 5 × 10^−08^) were reported.

### Protein-protein interaction network analysis

Proteins are involved in interacting directly and indirectly with other proteins and biomolecules to form scaffolds and complexes that eventually elicit the functional responses. Investigating these interactions can provide deeper understanding of the cellular process and functions associated with a gene or protein of interest^38^. In an effort to better understand the mechanistic and functional implications, SNPs that were successfully replicated and validated in previous steps were further investigated for protein interactions using STRING 10.5 (Search Tool for the Retrieval of Interacting Genes/Proteins)^39^. STRING database provides a score for each of the interactions indicating the estimated likelihood for biologically meaningful interactions based on the level and type of evidence. We only selected interactions with highest confidence score thresholds (STRING score >0.9) with combined evidence originating from text mining, experiments, databases and co-expression data. We report the most important biological process identified by STRING for the replicated and validated signals from our study.

### Ethics approval and consent to participate

The Institutional Review Boards at each of the participating sites reviewed and approved both PEAR and PEAR-2 study and written informed consents were acquired from all the participants.

## Supplementary information

Supplementary

## Data Availability

All the genotype and phenotype data supporting the conclusion of this article for both the studies, PEAR and PEAR-2 are available at dbGAP repository and can be accessed at (https://www.ncbi.nlm.nih.gov/projects/gap/cgi-bin/study.cgi?study_id=phs000649.v1.p1)0.20.The PEAR study data is available under dbGaP Accession: phs000649.v1.p1 and currently, the PEAR‐2 study data are in the process of being uploaded will soon be available under dbGaP Accession: phs000649.v2.p1.
