# Supplementary material for: Genome Wide Analysis Approach Suggests Chromosome 2 Locus to be Associated with Thiazide and Thiazide Like-Diuretics Blood Pressure Response
Source: Sci Rep. 2019 Nov 21;9:17323. doi: 10.1038/s41598-019-53345-5 (PMC6872535; doi:10.1038/s41598-019-53345-5)
Supplement: Supplementary file 1 — Supplementary [file 41598_2019_53345_MOESM1_ESM.docx]

**Genome Wide Analysis Approach Suggests Chromosome 2 Locus to be Associated with Thiazide and Thiazide Like-Diuretics Blood Pressure Response**

Sonal Singh(sonalsingh86@ufl.edu)^1^, Caitrin W. McDonough(cmcdonough@cop.ufl.edu)^1^, Yan Gong(gong@cop.ufl.edu)^1^, Kent R. Bailey(baileyk@mayo.edu)^2^, Eric Boerwinkle(Eric.Boerwinkle@uth.tmc.edu)^3^, Arlene B. Chapman(achapman1@medicine.bsd.uchicago.edu)^4^, John G. Gums(jgums@ufl.edu)^1^, Stephen T. Turner(sturner@mayo.edu)^5^, Rhonda M. Cooper-DeHoff(dehoff@cop.ufl.edu)^1,6^, *Julie A. Johnson(johnson@cop.ufl.edu)^1,6^

1 Department of Pharmacotherapy and Translational Research and Center for Pharmacogenomics, University of Florida, Gainesville, Florida, USA

2 Division of Biostatistics, Department of Health Sciences Research, Mayo Clinic, Rochester, Minnesota, USA

3 Human Genetics and Institute of Molecular Medicine, University of Texas Health Science Center, Houston, Texas, USA

4 Division of Nephrology, University of Chicago, Chicago, IL, USA

5 Division of Nephrology and Hypertension, Mayo Clinic, Rochester, Minnesota, USA

6 Division of Cardiovascular Medicine, Department of Medicine, University of Florida, Gainesville, Florida, USA

Supplementary Table 1. Independent SNPs with p < 1X10^-5^ for association with diastolic blood pressure response in African Americans

| **SNP** | **EFFECT ALLELE** | **MAF** | **P** | **BETA** | **SE** |
| --- | --- | --- | --- | --- | --- |
| 1:117288335 | A | 0.013 | 9.55E-06 | -19.26 | 4.19 |
| 1:229214289 | T | 0.019 | 9.87E-06 | -10.78 | 2.35 |
| 1:182097713 | C | 0.034 | 6.38E-06 | -9.18 | 1.96 |
| 1:110378181 | CT | 0.181 | 9.52E-06 | 4.15 | 0.90 |
| 1:23715978 | G | 0.205 | 8.30E-06 | 3.63 | 0.78 |
| 2:68350730 | T | 0.011 | 5.76E-06 | -15.75 | 3.34 |
| 2:158485292 | A | 0.013 | 1.26E-06 | -20.30 | 4.01 |
| 2:2870592 | G | 0.032 | 8.80E-06 | -8.85 | 1.92 |
| 3:113080567 | A | 0.015 | 9.99E-06 | -12.30 | 2.68 |
| 3:123014904 | T | 0.023 | 6.43E-06 | -12.66 | 2.70 |
| 3:47580813 | T | 0.123 | 9.19E-06 | -4.88 | 1.06 |
| 3:37843727 | AG | 0.241 | 6.77E-06 | -3.49 | 0.75 |
| 3:69281337 | G | 0.480 | 2.37E-06 | -3.59 | 0.73 |
| 4:180442771 | C | 0.024 | 4.15E-07 | -11.38 | 2.14 |
| 4:16303401 | G | 0.106 | 9.56E-06 | 4.12 | 0.90 |
| 4:187490357 | G | 0.427 | 3.54E-06 | -3.09 | 0.64 |
| 5:177754400 | C | 0.018 | 9.93E-06 | -12.89 | 2.81 |
| 5:10489128 | T | 0.019 | 4.19E-06 | -13.70 | 2.86 |
| 5:50624217 | G | 0.020 | 1.94E-07 | -12.50 | 2.28 |
| 5:10515903 | G | 0.035 | 1.81E-06 | -8.76 | 1.76 |
| 5:76449714 | G | 0.046 | 8.78E-06 | -7.86 | 1.70 |
| 5:58418501 | A | 0.085 | 2.55E-07 | 6.74 | 1.24 |
| 5:27411608 | T | 0.217 | 9.32E-06 | -3.91 | 0.85 |
| 6:162192205 | A | 0.017 | 2.82E-06 | -13.71 | 2.81 |
| 6:88202550 | A | 0.021 | 5.52E-06 | -10.39 | 2.20 |
| 7:10129002 | T | 0.011 | 1.33E-06 | -19.72 | 3.90 |
| 7:18282368 | A | 0.012 | 2.77E-06 | -15.18 | 3.11 |
| 7:144890144 | C | 0.026 | 3.99E-06 | -9.93 | 2.07 |
| 7:41513220 | C | 0.465 | 9.89E-06 | -3.49 | 0.76 |
| 8:89122108 | C | 0.014 | 1.26E-06 | -16.42 | 3.24 |
| 8:134743852 | T | 0.015 | 2.99E-06 | -14.94 | 3.07 |
| 8:81977545 | C | 0.016 | 1.01E-06 | -13.25 | 2.59 |
| 8:71365589 | T | 0.017 | 4.44E-06 | -13.25 | 2.77 |
| 8:92693591 | T | 0.034 | 8.60E-06 | -8.29 | 1.79 |
| 8:51443231 | C | 0.045 | 7.64E-06 | -7.21 | 1.55 |
| 8:142574962 | T | 0.184 | 6.16E-06 | -4.29 | 0.91 |
| 8:77343407 | G | 0.197 | 9.19E-06 | -3.96 | 0.86 |
| 9:31295759 | A | 0.027 | 2.52E-06 | -10.88 | 2.22 |
| 9:106700069 | T | 0.028 | 1.78E-07 | -10.07 | 1.83 |
| 9:115570712 | T | 0.062 | 5.14E-06 | -6.91 | 1.46 |
| 10:97987596 | C | 0.011 | 8.47E-06 | -15.92 | 3.44 |
| 10:33886562 | G | 0.112 | 5.78E-06 | 4.81 | 1.02 |
| 10:76827323 | A | 0.217 | 1.51E-06 | 3.84 | 0.76 |
| 10:76167912 | A | 0.271 | 9.19E-06 | 4.31 | 0.94 |
| 11:121771661 | A | 0.011 | 3.47E-06 | -13.12 | 2.71 |
| 11:60077367 | A | 0.011 | 9.66E-06 | -15.18 | 3.30 |
| 11:2913938 | T | 0.011 | 4.11E-07 | -18.32 | 3.45 |
| 11:2852309 | T | 0.027 | 7.91E-06 | -9.35 | 2.01 |
| 12:48800679 | A | 0.011 | 4.72E-06 | -17.06 | 3.58 |
| 12:119779963 | T | 0.015 | 2.94E-06 | -17.29 | 3.55 |
| 13:106475443 | T | 0.018 | 3.05E-07 | -12.60 | 2.34 |
| 13:76825276 | A | 0.024 | 6.27E-06 | -8.75 | 1.86 |
| 14:48940347 | T | 0.011 | 2.35E-07 | -18.36 | 3.38 |
| 14:36281438 | C | 0.013 | 4.07E-06 | -15.42 | 3.21 |
| 14:89369962 | A | 0.020 | 1.93E-06 | -9.68 | 1.95 |
| 14:62849226 | G | 0.055 | 4.21E-06 | -5.90 | 1.23 |
| 14:47049146 | G | 0.096 | 8.29E-06 | -5.28 | 1.14 |
| 15:24599180 | A | 0.028 | 7.41E-06 | -11.27 | 2.42 |
| 15:41901475 | C | 0.033 | 6.71E-06 | -8.51 | 1.82 |
| 15:63923093 | C | 0.092 | 4.53E-06 | -5.81 | 1.22 |
| 16:57510363 | A | 0.014 | 3.43E-06 | -14.31 | 2.96 |
| 16:77814065 | A | 0.031 | 6.70E-06 | -9.01 | 1.93 |
| 16:5571510 | T | 0.055 | 8.28E-06 | -7.05 | 1.52 |
| 16:13282923 | G | 0.241 | 9.81E-06 | 3.60 | 0.78 |
| 17:47989987 | G | 0.024 | 1.47E-06 | -13.08 | 2.60 |
| 19:42342073 | A | 0.026 | 2.74E-06 | -12.79 | 2.62 |
| 19:55129569 | AAAAAAAG | 0.072 | 9.26E-06 | 6.54 | 1.42 |
| 20:11017205 | C | 0.012 | 8.94E-06 | -18.31 | 3.97 |
| 21:45737605 | T | 0.026 | 9.92E-07 | -11.46 | 2.24 |
| 22:19321745 | A | 0.013 | 7.36E-06 | -15.60 | 3.35 |
| 22:41218683 | C | 0.019 | 5.36E-06 | -12.63 | 2.67 |
| 22:47691442 | T | 0.020 | 3.22E-06 | -13.15 | 2.71 |

SNP: chr:pos format; CHR: chromosome; MAF: Minor Allele Frequency; Beta: regression coefficient for allele Minor Allele; SE: Standard Error

Supplementary Table 2. Independent SNPs with p < 1X10^-5^ for association with systolic blood pressure response in the African American cohort

| **SNP** | **EFFECT ALLELE** | **MAF** | **P** | **BETA** | **SE** |
| --- | --- | --- | --- | --- | --- |
| 1:4550104 | A | 0.149 | 6.33E-06 | -5.93 | 1.26 |
| 1:28452553 | T | 0.019 | 5.79E-06 | 17.50 | 3.71 |
| 1:205796021 | C | 0.068 | 4.47E-06 | -10.53 | 2.21 |
| 1:218216863 | A | 0.028 | 1.10E-06 | 15.71 | 3.08 |
| 2:40645825 | A | 0.140 | 3.94E-06 | 6.55 | 1.36 |
| 2:60002943 | GC | 0.180 | 5.58E-06 | 5.86 | 1.24 |
| 3:39754820 | C | 0.359 | 7.29E-06 | -4.63 | 0.99 |
| 3:68603582 | G | 0.366 | 9.14E-06 | -4.52 | 0.98 |
| 3:70984708 | C | 0.025 | 6.71E-06 | 15.05 | 3.22 |
| 3:190369649 | G | 0.073 | 1.47E-06 | -11.49 | 2.28 |
| 4:140544936 | A | 0.290 | 3.36E-06 | 4.85 | 1.00 |
| 5:76411774 | T | 0.037 | 4.88E-06 | -12.72 | 2.68 |
| 5:156144905 | G | 0.012 | 3.65E-06 | 24.79 | 5.14 |
| 6:45490690 | T | 0.034 | 2.25E-06 | 14.74 | 2.99 |
| 6:158195695 | G | 0.288 | 4.18E-06 | -4.98 | 1.04 |
| 7:55729814 | C | 0.347 | 5.01E-06 | -4.86 | 1.02 |
| 7:133636770 | A | 0.032 | 7.84E-06 | 12.59 | 2.71 |
| 7:134039988 | A | 0.012 | 8.15E-06 | 20.96 | 4.52 |
| 7:137230391 | C | 0.031 | 4.38E-06 | 15.06 | 3.15 |
| 10:13468298 | G | 0.036 | 9.25E-06 | 12.73 | 2.76 |
| 11:21259199 | T | 0.257 | 3.54E-06 | 6.22 | 1.29 |
| 13:33831026 | A | 0.176 | 2.14E-06 | 6.24 | 1.26 |
| 13:92244167 | T | 0.031 | 4.78E-06 | 14.42 | 3.03 |
| 14:46568609 | A | 0.021 | 2.98E-06 | 21.21 | 4.36 |
| 14:68773739 | T | 0.244 | 3.45E-06 | 5.63 | 1.16 |
| 14:97468972 | C | 0.425 | 3.88E-06 | 5.02 | 1.04 |
| 15:42938962 | A | 0.367 | 6.68E-06 | -4.53 | 0.97 |
| 16:4200277 | T | 0.028 | 6.75E-06 | -15.14 | 3.24 |
| 16:7363944 | G | 0.027 | 9.62E-06 | 15.52 | 3.38 |
| 16:7504546 | G | 0.162 | 9.38E-06 | -6.53 | 1.42 |
| 17:70810958 | G | 0.057 | 8.58E-06 | 10.34 | 2.24 |
| 18:29665179 | G | 0.014 | 6.17E-06 | 21.45 | 4.57 |
| 20:52774427 | G | 0.409 | 7.11E-06 | 5.00 | 1.07 |
| 21:30014690 | A | 0.116 | 6.87E-06 | 7.09 | 1.52 |
| 22:19342329 | C | 0.017 | 1.99E-06 | -20.28 | 4.09 |

SNP: chr:pos format; CHR: chromosome; MAF: Minor Allele Frequency; Beta: regression coefficient for allele Minor Allele; SE: Standard Error

Supplementary Table 3. Independent SNPs with p < 1X10^-5^ for association with diastolic blood pressure response in the European American cohort

| **SNP** | **EFFECT ALLELE** | **MAF** | **P** | **BETA** | **SE** |
| --- | --- | --- | --- | --- | --- |
| 1:38527543 | C | 0.073 | 9.60E-06 | -4.44 | 0.97 |
| 1:115464269 | CA | 0.353 | 7.49E-06 | 2.78 | 0.60 |
| 1:212813455 | G | 0.021 | 9.96E-06 | -7.98 | 1.76 |
| 2:34319717 | A | 0.359 | 8.28E-06 | 2.20 | 0.48 |
| 2:148156515 | T | 0.029 | 6.74E-06 | -11.32 | 2.44 |
| 3:37276320 | T | 0.284 | 7.09E-06 | 2.61 | 0.56 |
| 3:65620508 | G | 0.025 | 8.35E-06 | -7.47 | 1.63 |
| 4:144288418 | A | 0.009 | 5.81E-06 | -13.28 | 2.84 |
| 5:1688900 | A | 0.041 | 2.93E-06 | -5.46 | 1.13 |
| 5:38751223 | C | 0.414 | 7.80E-06 | -2.19 | 0.48 |
| 6:26045367 | TTA | 0.045 | 2.16E-06 | -8.66 | 1.77 |
| 7:87570710 | A | 0.010 | 9.90E-06 | 18.71 | 4.11 |
| 9:18139412 | G | 0.235 | 7.44E-06 | 2.65 | 0.57 |
| 10:110176433 | C | 0.038 | 9.51E-06 | -6.30 | 1.38 |
| 11:127223995 | C | 0.377 | 6.32E-06 | -2.62 | 0.56 |
| 12:70421583 | C | 0.479 | 7.26E-07 | -2.67 | 0.52 |
| 14:95912388 | A | 0.076 | 9.01E-06 | -3.92 | 0.86 |
| 14:104359310 | T | 0.037 | 5.90E-06 | -8.10 | 1.74 |
| 15:38626475 | C | 0.102 | 7.76E-06 | -4.16 | 0.90 |
| 15:62437471 | C | 0.010 | 6.08E-06 | -14.23 | 3.05 |
| 16:56697867 | G | 0.126 | 2.75E-06 | -5.53 | 1.14 |
| 16:65929016 | G | 0.460 | 8.54E-06 | 2.20 | 0.48 |
| 17:42821246 | C | 0.010 | 4.83E-06 | -16.99 | 3.60 |
| 18:66520811 | AT | 0.024 | 2.79E-06 | 10.69 | 2.21 |
| 19:10408439 | C | 0.035 | 7.99E-06 | -8.44 | 1.84 |
| 20:10244172 | C | 0.041 | 4.21E-06 | -6.35 | 1.34 |
| 20:47032395 | A | 0.134 | 1.72E-06 | -5.42 | 1.10 |
| 20:61032694 | T | 0.081 | 6.44E-06 | 4.85 | 1.04 |
| 21:15364317 | A | 0.017 | 6.93E-06 | -13.57 | 2.93 |

SNP: chr:pos format; CHR: chromosome; MAF: Minor Allele Frequency; Beta: regression coefficient for allele Minor Allele; SE: Standard Error

Supplementary Table 4. Independent SNPs with p < 1X10^-5^ for association with systolic blood pressure response in the European American cohort

| **SNP** | **EFFECT ALLELE** | **MAF** | **P** | **BETA** | **SE** |
| --- | --- | --- | --- | --- | --- |
| 1:38527543 | C | 0.073 | 9.60E-06 | -4.44 | 0.97 |
| 1:115464269 | CA | 0.353 | 7.49E-06 | 2.78 | 0.60 |
| 1:212813455 | G | 0.021 | 9.96E-06 | -7.98 | 1.76 |
| 2:34319717 | A | 0.359 | 8.28E-06 | 2.20 | 0.48 |
| 2:148156515 | T | 0.029 | 6.74E-06 | -11.32 | 2.44 |
| 3:37276320 | T | 0.284 | 7.09E-06 | 2.61 | 0.56 |
| 3:65620508 | G | 0.025 | 8.35E-06 | -7.47 | 1.63 |
| 4:144288418 | A | 0.009 | 5.81E-06 | -13.28 | 2.84 |
| 5:1688900 | A | 0.041 | 2.93E-06 | -5.46 | 1.13 |
| 5:38751223 | C | 0.414 | 7.80E-06 | -2.19 | 0.48 |
| 6:26045367 | TTA | 0.045 | 2.16E-06 | -8.66 | 1.77 |
| 7:87570710 | A | 0.010 | 9.90E-06 | 18.71 | 4.11 |
| 9:18139412 | G | 0.235 | 7.44E-06 | 2.65 | 0.57 |
| 10:110176433 | C | 0.038 | 9.51E-06 | -6.30 | 1.38 |
| 11:127223995 | C | 0.377 | 6.32E-06 | -2.62 | 0.56 |
| 12:70421583 | C | 0.479 | 7.26E-07 | -2.67 | 0.52 |
| 14:95912388 | A | 0.076 | 9.01E-06 | -3.92 | 0.86 |
| 14:104359310 | T | 0.037 | 5.90E-06 | -8.10 | 1.74 |
| 15:38626475 | C | 0.102 | 7.76E-06 | -4.16 | 0.90 |
| 15:62437471 | C | 0.010 | 6.08E-06 | -14.23 | 3.05 |
| 16:56697867 | G | 0.126 | 2.75E-06 | -5.53 | 1.14 |
| 16:65929016 | G | 0.460 | 8.54E-06 | 2.20 | 0.48 |
| 17:42821246 | C | 0.010 | 4.83E-06 | -16.99 | 3.60 |
| 18:66520811 | AT | 0.024 | 2.79E-06 | 10.69 | 2.21 |
| 19:10408439 | C | 0.035 | 7.99E-06 | -8.44 | 1.84 |
| 20:10244172 | C | 0.041 | 4.21E-06 | -6.35 | 1.34 |
| 20:47032395 | A | 0.134 | 1.72E-06 | -5.42 | 1.10 |
| 20:61032694 | T | 0.081 | 6.44E-06 | 4.85 | 1.04 |
| 21:15364317 | A | 0.017 | 6.93E-06 | -13.57 | 2.93 |

SNP: chr:pos format; CHR: chromosome; MAF: Minor Allele Frequency; Beta: regression coefficient for allele Minor Allele; SE: Standard Error

Supplementary Table 5: Functional enrichment in the PPI network as identified by the STRING database for PPP3R1

| **GO:Term** | **Description** | **Count in Gene set** | **False Discovery Rate** |
| --- | --- | --- | --- |
| GO:0048016 | inositol phosphate-mediated signaling | 7 of 22 | 1.98X10^-15^ |
| GO:0033173 | calcineurin-NFAT signaling cascade | 6 of 10 | 1.99 X10^-14^ |
| GO:0019722 | calcium-mediated signaling | 7 of 132 | 5.31 X10^-11^ |
| GO:0038095 | Fc-epsilon receptor signaling pathway | 6 of 64 | 1.29 X10^-10^ |

GO: Gene Ontology; PPI: Protein-Protein Interaction

Supplementary Figure 1. Manhattan plots for association with BP response. Association of SBP and DBP in African Americans (a,b) and European Americans (c,d)


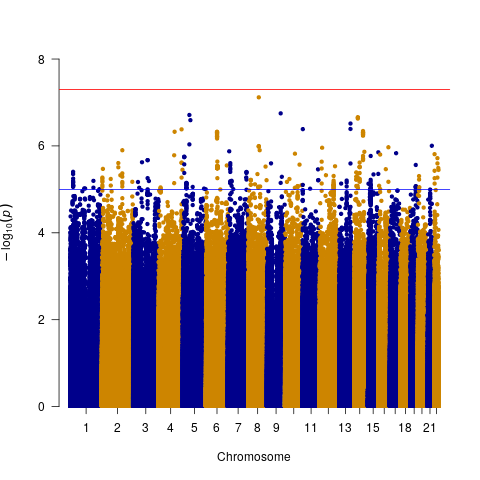

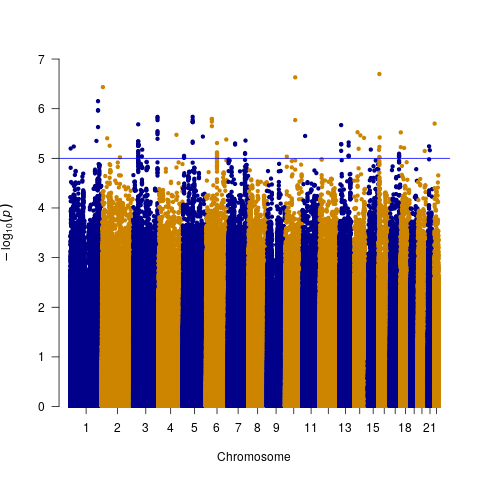

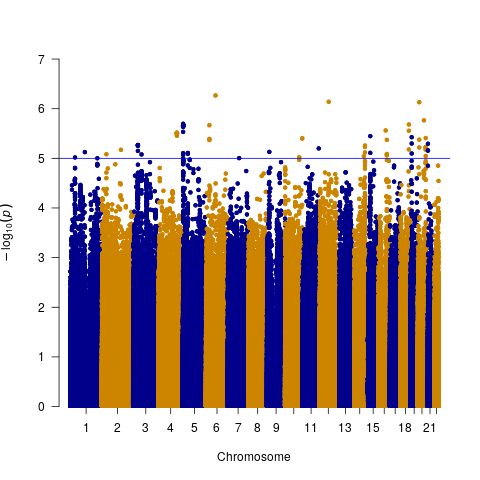

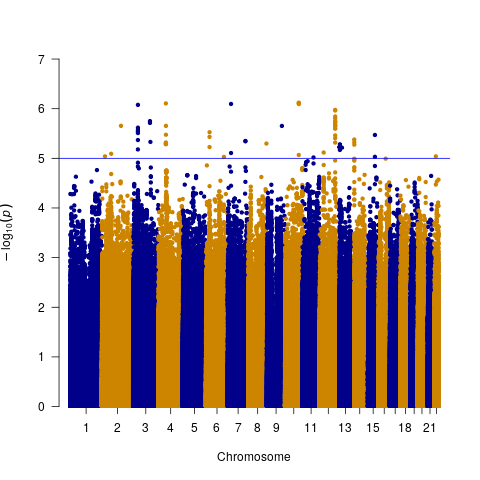


a

b

c

d


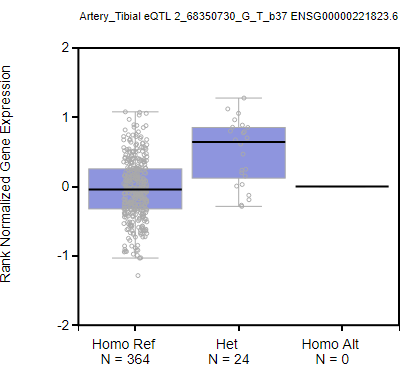


Supplementary Figure 2. eQTL of rs79237970 from GTEX database
